# The Development of Design and Manufacture Techniques for Bioresorbable Coronary Artery Stents

**DOI:** 10.3390/mi12080990

**Published:** 2021-08-20

**Authors:** Liang Wang, Li Jiao, Shuoshuo Pang, Pei Yan, Xibin Wang, Tianyang Qiu

**Affiliations:** 1 School of Mechanical Engineering, Beijing Institute of Technology, No. 5 Zhongguancun South Street, Haidian District, Beijing 100081, China; liangwangalex@outlook.com (L.W.); pang_shuo123@163.com (S.P.); 2 Key Laboratory of Fundamental Science for Advanced Machining Beijing Institute of Technology, No. 5 Zhongguancun South Street, Haidian District, Beijing 100081, China; jiaoli@bit.edu.cn (L.J.); pyan@bit.edu.cn (P.Y.); cutting0@bit.edu.cn (X.W.)

**Keywords:** bioresorbable stent, mechanical property, degradation behavior, biocompatibility, manufacture technique

## Abstract

Coronary artery disease (CAD) is the leading killer of humans worldwide. Bioresorbable polymeric stents have attracted a great deal of interest because they can treat CAD without producing long-term complications. Bioresorbable polymeric stents (BMSs) have undergone a sustainable revolution in terms of material processing, mechanical performance, biodegradability and manufacture techniques. Biodegradable polymers and copolymers have been widely studied as potential material candidates for bioresorbable stents. It is a great challenge to find a reasonable balance between the mechanical properties and degradation behavior of bioresorbable polymeric stents. Surface modification and drug-coating methods are generally used to improve biocompatibility and drug loading performance, which are decisive factors for the safety and efficacy of bioresorbable stents. Traditional stent manufacture techniques include etching, micro-electro discharge machining, electroforming, die-casting and laser cutting. The rapid development of 3D printing has brought continuous innovation and the wide application of biodegradable materials, which provides a novel technique for the additive manufacture of bioresorbable stents. This review aims to describe the problems regarding and the achievements of biodegradable stents from their birth to the present and discuss potential difficulties and challenges in the future.

## 1. Introduction

Vascular disease, including coronary atherosclerotic disease (CAD) and peripheral atherosclerotic disease (PAD), is a leading killer for humans in the world. Percutaneous coronary intervention (PCI) is a commonly used therapy for treating CAD and PAD. Balloon-expanded bare metal stents (BMSs) and drug eluting stents (DESs) are generally implanted with PCI to provide mechanical support for diseased arteries and prevent intimal hyperplasia [1]. After stent implantation, they are required to maintain good mechanical properties, biocompatibility, durability and corrosion resistance to allow for the recovery of the patient [2]. Previous clinical studies have shown that long-term complications (e.g., in-stent restenosis and thrombosis) have occurred after metallic stent implantation. Therefore, the development of stent design using materials with excellent biological and mechanical properties has become a top research topic in the biomedical and engineering fields. 

Bioresorbable stents (BRSs), the latest generation of stents, have advantages for replacing existing metallic stents because they can degrade and break down into natural by-products after fulfilling their intended purpose of providing sufficient support for diseased lesions [3]. The degradability of BRSs makes them available for patients of all ages, especially for children, due to their temporary implantation [4]. It is also possible for patients to accept further treatment in case of a second vascular disease incidence. The soft surfaces of BRSs are able to mitigate the damage caused by the contact of the vessel wall and the stent, which can decrease the adverse effects caused by cell attachment. Polymer coatings on BRSs are good containers for loading and releasing drugs into the human body that help restrain foreign body reactions. Consequently, BRSs have better biocompatibility compared with metallic stents due to the application of biomaterials including poly(l-lactic acid) and poly-ε-caprolactone [3,4]. 

Traditional stent manufacture techniques include etching, micro-electro discharge machining, electroforming, die-casting and laser cutting. In recent years, 3D printing technology has become a popular method for medical implant manufacture [5,6,7]. Fused deposition modeling (FDM), with the advantages of low cost, high reliability and simple operation, shows extensive potential for the large-scale production of cardiovascular stents. 3D printing technology can realize patient-specific customization, which means the process from medical image to stent production can be realized directly and quickly through 3D scanning techniques. In addition, the combination of smart materials with 3D printing can provide a novel technical solution for self-expanding stents with shape memory characteristics. 

This paper aims to review the comprehensive development of BRSs in terms of stent material and design, mechanical properties, degradation behavior, biocompatibility and manufacture techniques. Potential material candidates and design optimization methods will be presented. Mechanical property and degradation behavior studies will be classified. The advantages and disadvantages of existing stent manufacture techniques will be introduced and discussed. Finally, prospects and suggestions will be discussed for stent technology development.

## 2. Stent Material and Design

Stent material and design have a profound impact on the mechanical properties of vascular stents. Generally, stent design includes geometry and surface morphology. Stent geometry mainly affects radial properties, and surface morphology affects the interaction between stent and vessel, which may induce side effects.

### 2.1. Stent Design 

Abbott ABSORB 1.0 is the first available BRS to get a CE mark and FDA approval. The stent is made of poly(L-lactide) (PLLA) with a coating of poly (D, L-lactide) (PDLLA) and the antiproliferative drug everolimus. The crossing file is 1.4 mm, and strut thickness is 150 μm. The second generation ABSORB 1.1 has better radial support due to its optimized polymer processing and stent design [8]. There were 125,000 patients in 100 countries implanted with first- and second-generation ABSORB stents by March 2016 [8,9]. 

DESolve Nx stents (Elixir Medical Corporation, CA, US) obtained the CE mark in May 2014. DESolve stents use PLLA as their backbone material but have intrinsic self-correcting deployment properties, but ABSORB stents do not have [9]. The initial DESlove Nx stents have a crossing file of 1.5 mm and a strut thickness of 150 μm. The second-generation stents (DESolve 100) have a strut thickness of 100 μm [8]. DESolve Cx is another novolimus-eluting stent designed by Elixir Medical with a strut thickness of 120 μm. It is expected to provide enough mechanical support during vessel healing, and its safety and efficacy have been evaluated in six-month clinical reports [10].

The REVA stent (REVA Medical, San Diego, CA, US) is made of monomeric units of the common amino acid L-tyrosine and chemically modified by iodine, which can break down into carbon dioxide and water [11]. This stent has a crossing file of 1.8 mm and a strut thickness of 200 μm. The ReZolve stent, with the addition of a drug-eluting polymer, is an upgrade based on the REVA stent, and the ReZolve 2 stent is a sirolimus-eluting stent with a more extensive expansion range and higher radial strength [8]. The Fantom sirolimus-eluting stent is also based on the REVA stent and got its CE mark in April 2017 [8]; it is made of unique, proprietary iodinated struts with a thickness of 125 μm. The Fantom is intended to facilitate device delivery and precise target lesion treatment, and its 6-month clinical safety and efficacy is comparable to traditional metallic stents [12].

**Figure 1:**
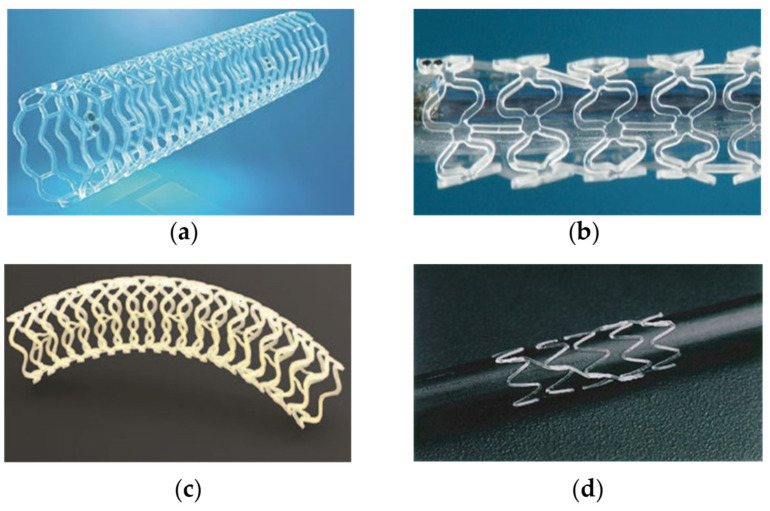
Bioresorbable stents: (**a**) Abbott ABSORB stent; (**b**) DESolve stent; (**c**) Fantom stent; (**d**) Igaki-Tamai stent.

The Igaki-Tamai stent is the first bioresorbable stent, which obtained its CE mark in November 2007. The stent diameters include 3.0, 3.5 and 4.0 mm with a length of 12 mm and a strut thickness of 170 μm. The stent is also made of PLLA but without a drug coating [8,13,14]. The stent was the first polymeric, self-expandable stent implanted in the human body with a zigzag helical coil design [14]. The long-term clinical trial results showed acceptable major adverse cardiac events, thrombosis rates and stent recoil [13]. The bioresorbable stents are showed in Figure 1.

The Magmaris™ (Biotronik AG, Buelach, Switzerland) is a metallic, sirolimus-eluting magnesium-based BRS with an open cell design. The square-shaped struts are 150 μm in thickness and 150 μm in width. The diameters of the scaffold sizes are 3.0 and 3.5 mm and the lengths are 15, 20 and 25 mm. Its nominal and burst pressures are 10 and 16 atmospheres (atm), and the diameter can be safely expanded up to a maximum of 0.6 mm. Only one thrombosis occurred in the early clinical trial, and a long-term clinical study is still needed [15].

### 2.2. Stent Material

BRSs are made of biodegradable polymers or corrodible metal alloys. Poly (lactic acid) (PLA), as one of the nontoxic and biocompatible polymers, has been widely used in medical implants such as sutures, tissue scaffolds, vascular grafts and vascular stents. This section aims to review existing and potential material candidates for bioresorbable stents.

**Figure 2:**
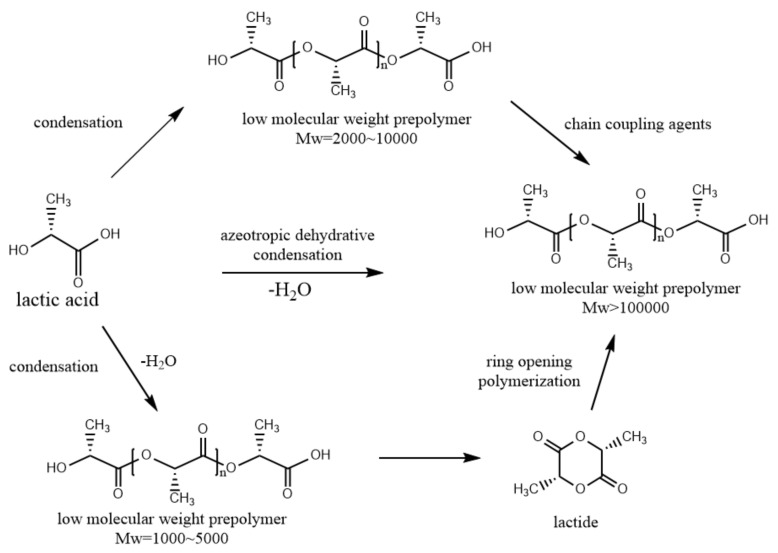
Synthesis of polymeric acid [18] (reproduced with permission from ref [18]; copyright 2001 Elsevier).

Swedish chemist Scheele first isolated PLA in sour milk in 1780. Lactic acid is prepared through commercial fermentation of potato and corn. It is commonly prepared in two ways, a solvent-based process or a solvent-free process [16]. Lactic acid is one of the chiral molecules and exists as two stereoisomers, L- and D-lactic acid. The plane rotates in a clockwise direction for L-lactic acid and rotates in an anticlockwise direction for D-lactic acid [17,18]. Polymerization of these two monomers forms three different types of lactide, namely L-lactide, D-lactide and meso-lactide. Therefore, PLA can exist in three different stereo-chemical forms: PLLA, PDLA and PDLLA. There are three synthesis methods for PLA, which are conventional polycondensation, dehydration condensation of lactic acids and ring-opening polymerization of lactides, as shown in Figure 2. The glass temperature and melt temperature of PLLA are about 55 °C and 175 °C, and the processing temperature is higher than 185–190 °C. Decreasing the melting point is the most common way to improve processing performance, but this significantly affects crystallinity and crystallization rates. PLLA can dissolve in some organic solvents such as tetrahydrofuran (THF), chlorinated solvents and benzene [19]. Many studies have been carried out to investigate the degradation behaviors of PLA and PLLA, and most of them have focused on analyzing their chemical properties, structure, thermal properties and mechanical performance for stent application [20,21,22,23].

There are also other polymeric materials used in the bioresorbable stents such as poly (lactide-co-glycolide) (PLGA), poly-glycolic acid (PGA), poly(D-lactide) (PDLA) and poly ε-caprolactone (PCL) [24,25]. 

PGA is the simplest linear aliphatic polyester, prepared by ring-opening polymerization of a cycle lactone, glycolide, with a crystallinity of 45–50% and a glass transition temperature of 35–40 °C. Due to its extraordinary mechanical properties, it is a suitable material for medical service [26,27]. However, PGA degrades very rapidly, leading to a loss of strength in 1–2 months, and its breakdown products cause inflammation in the surrounding tissues [28]. PGA was first used for bioresorbable sutures (DEXON) in 1960 due to its excellent processing capability [29]. Next, Terasaka et al. evaluated PGA nonwoven fabric composite efficacy as a novel biocompatible substitute [30].

PCL is a semi-crystalline linear polymer obtained from ring-opening polymerization of ε-caprolactone in the presence of a tin octoate catalyst [31]. Its glass transition temperature is low, at about −60 °C, and it is for this reason that PCL is often used as a compatibilizer or as a soft block in polyurethane formulations [27]. PCL mainly undergoes hydrolytic degradation because of hydrolytically labile aliphatic ester linkages, but it has a shorter degradation time (2–3 years) than PLLA due to its lower crystallinity degree [26]. Furthermore, it has been reported that PCL has a low tensile strength (about 23 MPa) but an extremely high elongation at break (>700%) [32]. The properties of the biodegradable polymers are summarized in Table 1 [33,34,35,36,37,38].

Magnesium alloy is a metallic material candidate for bioresorbable stents due to its low thrombogenicity and good biocompatibility. Pure magnesium degrades rapidly in aggressive chloride environments such as the human body, with various degradation rates over a range of 2 to 12 months through alloying with rare earth elements [39]. Thus, magnesium alloy can be an alternative material for bioresorbable stents.

## 3. Mechanical and Degradation Performance

### 3.1. Mechanical Performance

The mechanical properties of bioresorbable stents can be affected by their material properties and processing methods. Compared with metallic stents, bioresorbable stents do not have sufficient radial strength and stiffness, which may cause fracture and fatigue problems after stent implantation. In this part, we mainly introduce the mechanical properties of the stent, its impact factors and how to improve its mechanical properties.

Stent diameter and strut thickness are the most important characteristics when monitoring a stent’s mechanical performance in its development. Strut thickness bears the brunt among those factors. Stents with thinner struts can reduce restenosis effectively, and this has been validated in ISAR STEREO and ISAR STEREO 02 clinical trials. These trials carried out follow-up studies in 651 patients and 611 patients, respectively, and found that the thinner stent caused less angiographic and clinical restenosis than the thick stent [40,41].

Stent design is also a critical factor. Implantation of stents may create focal geometric irregularities related to strut protrusion. The protrusion of scaffold struts impacts local coronary flow dynamics, affecting endothelial shear stress (ESS) along the entire stent. ESS is derived from the friction of flowing blood on the endothelial surface [42]. Emerging studies have proven that low ESS increases scaffold restenosis and thrombosis [43,44,45,46,47]. The relationship between protrusion distance and shear stress is influenced by stent geometry. Bourantas et al. investigated the relationship by following 12 patients with implanted ABSORB bioresorbable vascular scaffolds for one year. Results showed that the ESS impact on vessels needed to be seriously considered when designing stents [48]. However, most current experiments have been carried out by considering healthy coronary arteries. There are still no sufficient studies that examine strut protrusion and stent composition, which influence blood flow hemodynamics [49]. Analyzing protrusion can guide stent designs and determine hemodynamic performance, which dramatically impacts stent development. 

The collapse pressure is an essential factor that can reflect mechanical performance. Previous research has shown that the surface area of a stent has a significant effect on the collapse pressure. A large surface area benefits load bearing when using the same material. The material molecular weight does not have such an effect and affects neither the tensile strength nor Young’s modulus [50]. Collapse pressure should be considered in the study of stent degradation performance. The collapse pressure changes sensitively during material degradation, which will be discussed in Section 3.2. 

Stent recoil is generally used as a comparable parameter when evaluating the expansion behavior of stents. Elastic recoil resists blood flow and increases the risk of restenosis. The recoil hinders the tissue’s healing procedures and creates a blockage in the blood vessels because the stent needs to have a certain degree of self-expandability, either anchoring more easily against the blood vessel wall or counteracting the recoil [51]. The control of stent recoil needs to balance the stent’s geometry and the stent’s materials.

Researchers have made great contributions toward improving the mechanical properties of bioresorbable stents, and plasticizing has proven to be an effective method. Previous research has shown that PLLA containing less than 5% triethyl citrate (TEC) as a plasticizer makes the stents obtain higher creep resistance and sufficient elongation at break [52]. 

The crystallinity and molecular weight of a stent’s material can be strongly affected by material processing, sterilization and annealing [53]. Therefore, it is vital to choose the proper way to sterilize a stent without infecting its mechanical performance. Steam sterilization is commonly used for medical implants, and results showed that temperatures over 100 °C can decrease molecular weight but increase the elastic modulus [54]. Ethylene oxide and γ-irradiation are also used as sterilization techniques. γ-irradiation can reduce molecular weight and strength and break down fiber structure, which leads to the weakening of mechanical properties. On the contrary, ethylene oxide sterilization seems to have little effect on the mechanical behavior of PLLA [55]. However, ethylene oxide creates toxic residues in the polymer due to its lengthy degassing procedure [54]. 

The most common processing of poly (lactic acid) is through injection and extrusion/injection. The chain scission can decrease the molecule weight of the material and the elongation of the injected PLLA can be improved through chain reprocessing. The annealing process can increase the crystallinity of polymeric materials, and further strengthen the Young’s modulus and the yield stress [56]. 

A researcher also found that thinning a strut directly could improve its radial strength. The stents were divided into two groups, commonly stretched stents and thinned stents. Having conducted a three-stage tensile strength trial, the researcher demonstrated that the thinned stents had better radial strength [57]. This work gives a new perspective to stent development.

### 3.2. Degradation Properties

Bioresorbable polymers can break down in biomedical environments. PLLA is widely used in medical service, including in bioresorbable stents and sutures [58]. Poly (lactic acid) in its L and D forms has been proven to be safe and effective in the human body. PLLA usually takes four months to ten years to degrade [59]. PLLA generally degrades when its ester bonds hydrolyze into lactic acid, which is metabolized from the body [60]. The degradation rate of PLLA is affected by its molecular weight, crystallinity and degradation environment. 

Many factors affect the degradation behavior of polymeric stents including time, temperature, molecular weight and catalyst concentration. The rate of degradation depends on size, structure and temperature [19]. Polymeric stent degradation usually consists of three stages: Firstly, the polymer absorbs water, which cuts the long chemical bond chains into many short chains. Short chains break down into monomers, and molecular weight begins to decrease during this stage. Secondly, because the short chains have less mechanical energy, chains break down more easily and decrease the strength of the polymer. Finally, the polymers begin to lose all mass and structure and are finally broken down [3]. Stents with higher molecular weights can benefit cell attachment and proliferation. 

Cell attachment establishes an interaction relationship, which promotes cell growth. Stent degradation can decrease pH, which also affects cell proliferation [61]. Naseem et al. used atomic force microscopy and nanoindentation techniques to analyze the mechanical performance of stents during two years of degradation in vitro. Nanoindentation showed advantages for monitoring of the change of Young’s modulus compared to atomic force microscopy [62].

The degradation rate and mechanical performance of PLLA can be modified through the addition of ingredients. Researchers investigated the degradation behavior of PLLA/PCL blends with different weight ratios of 100/0, 80/20, 60/40, 40/60, 20/80 and 0/100. Results showed that PLLA/PCL (80/20) exhibits an accelerated degradation rate as well as greater impact strength [63,64]. Bobel et al. studied the stress–strain, recovery, relaxation and creep behavior of PLLA stents at body temperature. They also evaluated the pre-degradation of PLLA stents and the feasibility of PLLA as a stent material candidate [65].

It is essential to consider the effects of material degradation performance in vivo when designing and analyzing bioresorbable stents. In order to capture the degradation process of stents, computational modeling techniques have been developed [66,67]. Phenomenological modeling has been applied to examine the degradation behavior of bioresorbable stents. Rajagopal et al. introduced a stain-induced model consisting of thermodynamics and a polymer chain scission while measuring the extent of local degradation [68,69]. Soares et al. developed this theory, mainly focusing on PLLA stents [70].

Soares et al. believe that a change’s degradation rate is related to applied strain, current degradation state, spatial location and time. The relationship among them is:

(1) $$\;\frac{d}{dt}d\left(t\right)=C\left(1-d\left(t\right)\right){\left[{\left(I_{1}-3\right)}^{2}+{\left(I_{2}-3\right)}^{2}\right]}^{\frac{1}{2}}$$

where *C* is a time constant and *I_1_* and *I_2_* are the first and second strain invariants. In a constitutive model of the material, the strain energy is relevant to the degradation parameter through damage-based evolution of the shear modulus [60,71]. The relationship between shear modulus *μ* and initial shear modulus *μ_0_* is:

(2) $$\;\;\mu =\mu_{0}\left(1-d\right)$$

Muliana and Rajagopal examined the effects of viscoelasticity and water diffusion on degradation. They chose the quasi-linear viscoelastic (QLV) constitutive model to predict the time-dependent mechanical response of polymeric stents. They also examined the effect of the coupling response between the polymeric stent and the arterial wall on the degradation of biodegradable polymeric stents [72]. Luo et al. established a numerical model with user-defined field variables to examine the degradation performance of cardiovascular stents. In vitro and in vivo tests can provide physical insights and predict stent degradation performance [73]. Shazly et al. developed an integrated computational model that could predict the bulk degradation and by-product fate of PLLA stents. They evaluated the relative impacts of PLLA degradation rate, arterial remodeling and metabolic activity on local lactic acid [74]. Khan and El-sayed developed a constitutive model that combined Maxwell- and Ogden-type models. This model, when integrated with finite element software, can predict the time-dependent response of a biodegradable stent subjected to finite deformation and under complex mechanical loading conditions [75]. Lin et al. developed a strain-based degradation model to estimate the dynamic interactions between the stent and the artery. The model obtained a nonlinear relationship between the maximum principal strain of the stent and the fracture time that can predict the degradation process under different mechanical conditions [76]. 

### 3.3. Clinical Trial

Ormiston et al. evaluated the everolimus-eluting stent produced by Abbott Vascular. The trial chose 30 patients from four centers: Auckland, Rotterdam, Krakow and Skejby. The clinical endpoints were cardiac death, myocardial infarction and ischemia-driven target lesion revascularization. Angiographic and intravascular ultrasounds were used to evaluate clinical outcomes at 6 and 12 months after implantation. The results showed a small in-stent loss and a neointimal area at six months. There is one patient presented with non-Q wave myocardial infarction at the one-year assessment, and the clinical trial results showed that the adverse event rate was 3.3% [77]. Serruys et al. compared an everolimus-eluting bioresorbable scaffold (Absorb, Abbott Vascular, Santa Clara, CA, USA) with an everolimus-eluting metallic stent (Xience, Abbott Vascular, Santa Clara, CA, USA). Three hundred thirty-five patients were implanted with bioresorbable scaffolds, and 166 patients were implanted with metallic stents. There were 17 major cardiac adverse events in the bioresorbable scaffold group and 3 in the metallic scaffold group. The most common adverse events were myocardial infarction and target lesion revascularization. The bioresorbable stents showed similar clinical results as the metallic stents [78]. In 2018, Stone et al. conducted a randomized ABSORB IV trial in which patients had stable coronary artery disease or acute coronary syndromes. One thousand, two hundred ninety-six patients were implanted with BVSs, and 1308 patients were implanted with Xience stents. After 30-day and 1-year evaluations, target lesion failures and angina rates were similar between the two groups. However, in the BVS group, adverse events happened more than in the other group, which demonstrated that the BVSs needed further improvements [79]. Muramatsu et al. also performed an ABSORB-EXTEND single-arm trial in 2013 and got similar results [80]. 

The Igaki-Tamai stent is another remarkable PLLA stent that was implanted into the human body to evaluate its safety and efficacy. Fifteen patients were successfully implanted with 25 stents. Coronary angiography and intravascular ultrasound were applied to access the safety and efficacy of stents at one day, three months, and six months. There is no significant recoil or significant stent expansion observed by ultrasound. At six months, the restenosis rate and target lesion revascularization rate were 6.7% per patient and 10.5% per lesion. Other than repeat angioplasty, no major cardiac reverse event occurred [14]. The scientists followed the patients for 10 years of major cardiac events and scaffold thrombosis rates. The rates of all-cause death, cardiac death and major adverse cardiac events over 10 years were 87%, 98% and 50%. The cumulative rates of target lesion revascularization (target vessel revascularization) were 16% (16%) at 1 year, 18% (22%) at 5 years and 28% (38%) at 10 years [13]. The clinical results showed the long-term safety and efficacy of PLLA stents.

The NeoVas is a sirolimus-eluting stent produced by Lepu Medical. Two hundred seventy-eight patients were chosen in the RCT trial and 825 patients in the registry trial. Target lesion failure and the patient-oriented composite endpoint were analyzed by 12 months, which suggested that the stent was safe and effective in the human body. The biocompatibility of the stent was also evaluated in porcine coronary arteries [81]. Feng et al. added nano-amorphous calcium phosphate (ACP) into a PLLA stent to improve the mechanical support of the scaffolds. They implanted the PLLA/ACP stents and PLLA stents into human bodies. After 1 month, 6 months, 12 months and 24 months of monitoring, PLLA/ACP stents were proven to be reliable and biocompatible [82]. 

XINSORB is the first commercial stent in China, made by Huaan Biotechnology Group. The resorption time of the stent is 24–36 months and the stent strut thickness and the stent diameter are 160 μm and 3.0 mm. XINSORB stents were implanted in 30 patients with a 100% success procedure rate from September 2013 to January 2014. The endpoint of TLF occurred in 4 patients, and 5 patients experienced major cardiac events. There were no more cases that occurred after two years of follow-up, and the clinical endpoints had no changes after three years [83]. 

Firesorb is a new generation product designed by MicroPort, Shanghai, China, consisting of a PLLA backbone and coated with PDLLA and sirolimus. Forty-five patients were chosen in the FUTURE I study to evaluate the safety and feasibility of the stents. Patients were divided into two groups, and the examinations (angiographic, IVUS or OCT) were carried out at different time points. After four years of follow-up, only two patients suffered patient-oriented composite endpoints (PoCEs), and no scaffold thrombosis or TLF events were observed. The implanted stents showed completed absorption during the 4-year trial, which demonstrated the effectiveness of the stents [84].

## 4. Biocompatibility

Biocompatibility is a key parameter that needs to be considered for medical devices, especially for coronary stents [85]. The bioresorbable materials that we have mentioned are biocompatible but still have some biocompatibility problems. Reports have shown that bioresorbable implants may cause different adverse effects and need reassessment [86,87]. Bioresorbable implants may cause foreign body reactions, immunological reactions, allergies and inflammatory responses due to material composition, degradation process, device shape and size. [88,89,90,91,92]. For coronary stents, contact with the vessel wall damages the endothelial vascular tissue, which induces an inflammatory reaction and then causes restenosis [93,94]. In those cases, medical diagnosing methods including angiography and intravascular ultrasound are required to monitor clinical outcomes after stent implantation. 

Van der Giessen et al. investigated the biocompatibility of five biodegradable polymeric stents: PGA/PLA, PCL, polyhydroxy butyrate valerate, poly-orthoester and polyethylene oxide/polybutylene terephthalate. Severe inflammatory responses were observed in all cases, and potential reasons included stent design, stent material and the sterilization process [95]. Sterilized PLLA stents were implanted into porcine femoral arteries and inflammation problems were also reported, probably due to the raw material formulation [96]. Three polymeric stents, including a PLLA fiber stent, a PLLA stent and a PLLA/PDLA stent, were implanted in animal models for a biocompatibility study. The 24-month follow-up reports showed that the lowest inflammation response occurred in PLLA/PDLA stent cases, and suggested that PLLA/PDLA can be a stent material candidate [37,97,98].

### 4.1. Surface Modification

There are some useful methods for improving the biocompatibility of coronary stents such as surface modification and drug coating. Surface modifications can benefit the recovery of damaged vascular walls and enhance endothelial cell migration, anchorage and proliferation [99,100,101,102,103]. In general, biocompatibility is highly correlated with surface properties and interactions between the stent surface and endothelial cells or proteins [37,101,102,103,104,105,106,107]. There are specific proteins that should be considered in clinical assessments of blood-contacting devices, especially for coronary stents. Albumin can decrease platelet adhesion and binding of microorganisms, which may cause severe infection. Fibrinogen and immunoglobulin G (IgG) can instigate a host response toward increased platelet adhesion [98]. There are many surface modification methods available for stent application to improve blood compatibility and re-endothelializiation [99,108,109], as shown in Table 2.

Extruded PLLA with curcumin can reduce the inflammatory response effectively. Stents with porous surfaces can enhance surface function to obtain better biocompatibility [156]. Rudolph et al. performed different surface modification techniques on five different polymers to evaluate their biocompatibility, including wet chemical (NaOH and ethylenediamine) and plasma chemical (O_2_ and NH_3_) processing methods. Results showed that the modified polymers exhibited better biocompatibility than the unmodified polymers, and the NH_3_ plasma-modified polymers were significantly enhanced in terms of cell viability, adhesion and morphology [157]. Lee et al. fabricated a PLLA biodegradable stent through 3D printing and performed surface modification with polydopamine (PDA), polyethyleneimine (PEI) and heparin (HEP). The biocompatibility assessment results indicated that the modified PLLA stents exhibited good blood compatibility and showed advantages in preventing restenosis and thrombosis with anticoagulation [158].

### 4.2. Drug Coating

Drug-eluting stents have experienced significant development in terms of drug categories and drug delivery mediums [4]. Sirolimus is one of the drugs widely used on coronary stents, and previous research has shown that sirolimus release can inhibit smooth muscle cell proliferation restenosis and neointimal hyperplasia. The release kinetics of sirolimus has mainly been investigated by means of monitoring the PBS release in vitro, but the stability cannot be guaranteed. Naseerali et al. formulated a novel medium of normal saline and isopropanol (9:1) to access the release kinetics of sirolimus, and its efficacy was verified in trials [159]. The drug release rate is a major parameter for evaluating the performance of drug-eluting stents. The drug release process can be divided into three stages: At first, the drug release rate is relatively fast, it then becomes slow, and then it finally achieves saturation. A faster release rate may lead to several adverse effects (i.e., delayed endothelialization). The initial release rate is generally correlated with the doses of sirolimus and the coating medium (i.e., PEG). These coating mediums have been proven to be effective for optimizing drug release kinetics [160]. Sirolimus-coated stents are summarized in Table 3 [8].

Stents coated with PLLA and genistein can reduce the risk of restenosis after implantation [161]. Tyrosine kinase inhibitor ST638 was loaded onto stents and implanted into pig models; the results showed that it could reduce restenosis and suppress proliferative stimulation [162]. Nanoparticles are also used as coating materials for stents, and results have shown that stents coated with PDLLA nanoparticles and sirolimus exhibit relatively slow drug release rates. In addition, PDLLA coatings can help restrain the proliferation of smooth muscle cells and promote endothelial cells’ proliferation [163].

Recombinant polyethylene glycol (r-PEG)-hirudin and the prostacyclin analog iloprost are also effective for reducing adverse reactions. Standard pressure-coated stents were implanted in sheep as well as overstretched models in pigs for about 28 days. The results showed that the restenosis areas of the sheep-coated group and the pig-coated group decreased by 22.9% and 24.8% without increasing other inflammatory responses [164]. Lincoff et al. used dexamethasone as a drug coating on PLLA stents and figured out that inflammatory responses were mitigated in the cases of PLLA stents with low molecular weights [165,166].

Lactic acid (LA) is the first degradation product of PLLA. It induces the endothelial-to-mesenchymal transition (EndMT) through the TGF–β1 pathway. In this way, LA may induce vascular fibrosis, which may cause severe in-stent stenosis. Moreover, PLLA degradation may also cause inflammation in aortic endothelial cells [167]. Curcumin has been found to be effective for anti-inflammatory and endothelial dysfunction. Previous studies have shown that it can decrease the risk of thrombosis in animal trials and h reduce thrombosis rates in human arteries [168].

## 5. Stent Manufacture Techniques

Stent manufacture techniques have undergone a development process with continuous breakthroughs and progress. There are five existing stent manufacture techniques: etching, micro-electro discharge machining, electroforming, die-casting and, most commonly used, laser cutting [169].

**Figure 3:**
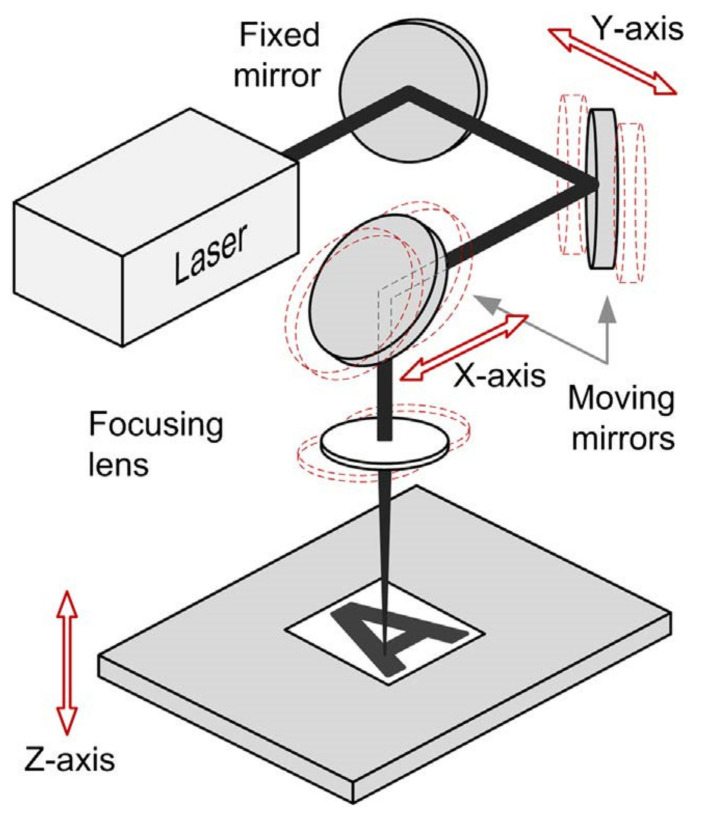
Direct-write CO_2_ laser micromachine system [170].

Stepak et al. used a CO_2_ laser to fabricate PLLA stents, and the processing quality results suggested that laser cutting can be an alternative technique for stent manufacture [170]. The CO_2_ laser cutting system is shown in Figure 3. Guerra et al. reported that the laser-cut PCL stent obtained a dimensional precision of 95.75% [171]. Tamrin et al. figured out that laser power has an effect on the heat zone for all thermoplastics [172]. Although laser cutting is a relatively mature and widely used stent manufacturing technology, it still has some shortcomings. First of all, for workpieces with narrow dimensions such as stents, excessive heat during laser processing will have a relatively significant impact on the mechanical performance of the stent. Secondly, laser cutting stents cannot meet the individualized customization needs of patients due to their processing principles. This is not conducive to the recovery of some special populations such as pediatric patients. Finally, the cost of laser cutting is higher, and the time cost of mass production is still high. This has made people focus on 3D printing technology with high economic efficiency, personalized customization and more diversified material selection.

**Figure 4:**
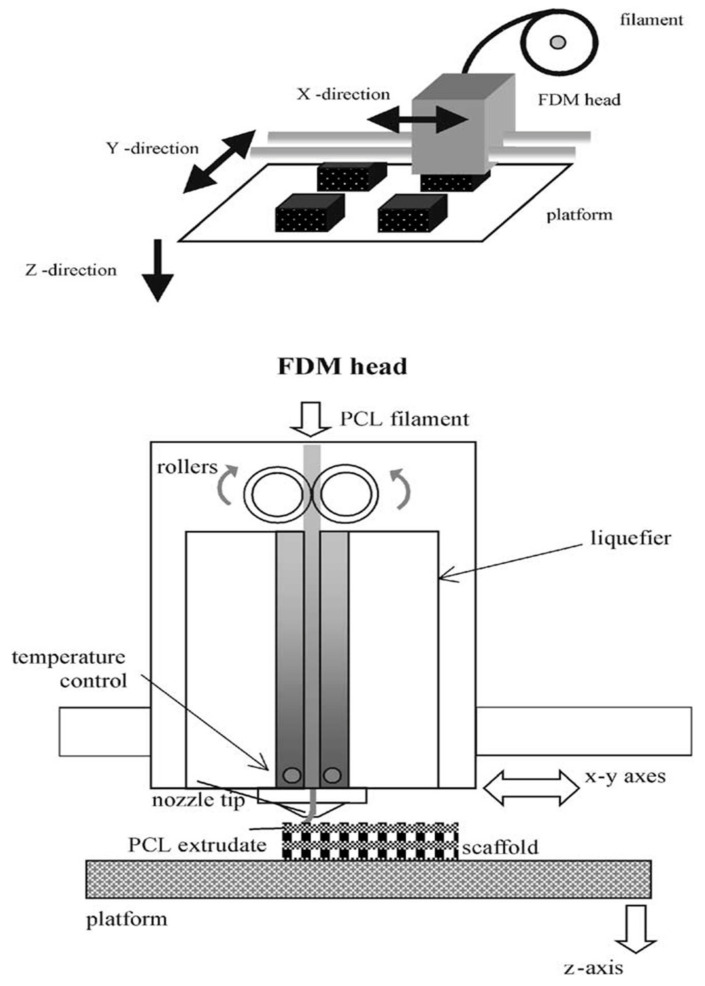
Extrusion and deposition processes Schematic diagram of FDM [173] (reproduced with permission from ref [173]; copyright 2001 Elsevier).

3D printing technology types include inkjet, stereolithography, selective laser sintering and fused deposition modeling (FDM) (as shown in Figure 4). With the continuous development of 3D printing technology, it has been widely used in medical fields.

Most degradable stent materials are not suitable for traditional processing methods, so biological 3D printing technology has attracted a great deal of interest and attention for stent manufacture.

Guerra et al. designed a novel, 3D additive manufacturing machine to produce stents and studied the effects of nozzle temperature, fluid flow and printing speed on the geometrical features of stents. Results showed that printing precision is highly affected by nozzle temperature and fluid flow. Furthermore, they developed a dimensional prediction model to improve dimensional precision [174]. Wang et al. developed a new screw extrusion-based 3D printing system for stent fabrication, especially by designing a zero-Poisson’s ratio structure [175].

Park et al. fabricated a bioabsorbable stent prototype using 3D printing technology. The fabricated stent was coated with a mixture of sirolimus and poly-(lactide-co-glycolide) (PLGA) and polyethylene glycol (PEG) to decrease the drug release rate. The kinetics of sirolimus exhibits a sustained release profile, and can help reduce neointimal hyperplasia [5]. Qiu et al. developed a rotary 3D printing method for PCL polymer stents. 2-N,6-O-sulfated chitosan (26SCS) was used to modify the stents’ characterization by the surface microstructure. The PCL stents and modified PCL stents showed excellent biocompatibility, and the modified stents enhanced cell proliferation. There was no significant difference in mechanical behavior between these two stents [176]. Guerra et al. first used a composition of PCL and PLA to fabricate stents by means of 3D printing technology. The mechanical and degradation performance of stents were evaluated by cell proliferation, degradation, dynamic mechanical and radial expansion experiments. The results demonstrated that composite stents with 3D printing technology are likely to overcome the complications of polymeric stents [177]. Wu et al. manufactured PLA stents with an arrowhead negative Poisson’s ratio design using fused deposition modeling. The results showed that the radial force of a PLA stent can be improved by increasing the wall thickness and the surface coverage, decreasing the stent diameter. More importantly, the radial and longitudinal size of the stent crimp under deformation temperature and expand at recovery temperature. This phenomenon demonstrates the feasibility of the shape memory effect of PLA as a reliable basic material for 3D printing stents [178]. Researchers developed a novel biodegradable polymer–graphene composite with dual drug incorporation. They directedly fabricated stents from medical images using 3D printing, which fulfilled personalized demands. Both physical and chemical properties of the stents were investigated, and results suggested that the stents were safe in pigs’ coronary arteries. The blood pressure and blood flow were predicted, and the compression capacity of stent was optimized by in silico analysis [179].

## 6. Conclusions and Future Work

The current generation of biodegradable stents has undergone significant development in terms of material processing, design optimization and manufacture techniques, but further work is still required to improve their clinical safety and efficacy. Bioresorbable stents have benefits compared to metallic stent sin current stent technology development. However, due to a series of side effects after stent implantation such as thrombus, in-stent restenosis and inflammation, almost all degradable stents on the market have been discontinued or removed from the shelves. Further improvement of the degradable stent can be carried out as follows:

1. The development of biodegradable materials plays an important role in the development of biodegradable scaffolds. Compared with metallic scaffolds, biodegradable scaffolds still have many deficiencies in radial strength and other mechanical properties that need to be continuously improved upon and developed in the future. Good mechanical properties can prolong the service life of a stent and provide strong support at the lesion and injury site, which is conducive to the recovery of patients. The material processing method can significantly influence the mechanical properties of the scaffold. Exploring new stent processing methods has become a hot research direction;
2. The degradability of bioresorbable scaffolds is also a key property characteristic. The assessment of degradation performance is generally divided into in vivo and in vitro degradation experiments. In vitro degradation experiments are usually conducted in pH- and temperature-specific solutions such as tetrahydrofuran solution with a pH of 7 at 37 °C. In vivo degradation experiments can also be divided into animal experiments and human experiments. Animal studies have been carried out in rabbits, mice, pigs and sheep to assess whether stents cause severe inflammation and cellular problems. Similarly, stents can be implanted in humans to evaluate their six-months, one-year or long-term performance after implantation.
3. Excellent biocompatibility plays a vital role in the development of medical implants. Stents come into direct contact with the cardiovascular and blood vessels after implantation, which is a major cause of clinical complications. Surface modification and drug coating of scaffolds should be proposed to improve stent biocompatibility. The surface texture of the scaffold can be modified using physical and chemical methods to intuitively reduce the contact between the scaffold and the blood vessels. Stent drug loading can reduce complications and control the degradation rate of stents through drug release, which is also the direction of future development.
4. Additive manufacturing has been a hot topic in the medical field in recent years. Many studies have shown that 3D printing may be an alternative scaffold fabrication method through developing intelligent polymer materials. Shape memory materials are also a new development direction. The development of biodegradable stents with shape memory performance can simplify the complex process of stent implantation and provide more convenient services for doctors and patients.

## Figures and Tables

**Table 1:** The properties of the biodegradable polymers.

Polymer	Tensile Strength (MPa)	Young’s Modulus (GP)	Yield Strength (MPa)	Melting Point (°C)	Elongation (%)
PLA	21–60	4.0	70	150–162	4
PLLA	45–70	2.17	57	173–178	3.3
PDLA	46	2.16	46	Amorphous	2.6
PDLLA	40	1-3	-	Amorphous	-
PGA	77.3	3.33	77.3	220–225	3.9
PCL	20–35	0.4	-	58–63	-

**Table 2:** Surface modification methods.

Methods	Principle	Function
Surface roughening [104,110,111,112,113,114,115]	Oxygen plasma deposition Argon plasma deposition Etching Sanding	Decrease cell migration No chemical alteration Increase surface area Restrict cell movement Enhance cell attachment
Surface patterning [102,114,116,117,118,119,120,121,122,123,124,125,126,127,128,129]	Lithography Microfluidic Self-assembled Monolayers Transfer printing Stencil-assisted printing Nanopatterning	Quell non-specific protein–surface interactions Enhance endothelial cell attachment Encourage vessel healing Promote anti-thrombotic properties
Chemical modification [99,104,121,130,131,132,133,134,135,136,137,138]	Chemical vapor deposition Plasma vapor deposition Grafting techniques Self-assembled monolayers	Enhance the functionality of the surface
Surface coatings and films [139,140,141,142,143]	Wet/solvent coating Langmuir-Blodgett films	Increase endothelial cell attachment Reduce blood coagulation and thrombosis
Attachment of pharmaceuticals or biopharmaceuticals to the surface [104,131,144,145,146,147,148,149,150,151]	Chemical vapor deposition Wet chemical surface modification Plasma treatment Nitric oxide or thrombomodulin Layer by layer Polypyrrole composites	Control cell behavior Direct cell signaling
Porous surfaces to facilitate drug delivery [152,153,154,155]	Drugs attached directly	Stimulate vessel healing Better incorporation with body

**Table 3:** Stents coated with sirolimus.

Company	Stent	Base Material	Strut Thickness (µm)	Stent Diameter (mm)	Degradation Time (Months)	Polymer-Based Coating
Meril Medical	MeRes	PLLA	>200	3.0	24	-
	MeRes	PLLA	100	2.5, 3.0, 3.5	-	PDLLA
Amaranth	FORTITUDE	PLLA	150–200	2.75	3–6	-
Huaan Biotechnology Group	XINSORB	PLLA	160	3.0	24–36	PDLLA/PLLA
Manli Cardiology	Mirage	PLLA	125–150	3.0-3.5	14	PLA
Arterius	ArterioSorb 120	PLLA	120	-	-	PDLA

## Data Availability

Not applicable.
